# Ventricular arrhythmias in acute heart failure: a clinical consensus statement of the Association for Acute CardioVascular Care, the European Heart Rhythm Association, and the Heart Failure Association of the European Society of Cardiology

**DOI:** 10.1093/europace/euae235

**Published:** 2024-09-13

**Authors:** Bulent Gorenek, Adrianus P Wijnmaalen, Andreas Goette, Gurbet Ozge Mert, Bradley Porter, Finn Gustafsson, Gheorghe-Andrei Dan, Joris Ector, Markus Stuehlinger, Michael Spartalis, Nils Gosau, Offer Amir, Ovidiu Chioncel

**Affiliations:** Eskisehir Osmangazi University, Faculty of Medicine, Department of Cardiology, ESOGÜ Meselik Kampüsü, Büyükdere Mahallesi, Prof. Dr Nabi AVCI Bulvarı No: 4 Odunpazarı, Eskisehir 26040, Turkey; Department of Cardiology, Leiden University Medical Center, Leiden, Netherlands; Department of Cardiology, Saint Vincenz Hospital Paderborn, Paderborn, Germany; Eskisehir Osmangazi University, Faculty of Medicine, Department of Cardiology, ESOGÜ Meselik Kampüsü, Büyükdere Mahallesi, Prof. Dr Nabi AVCI Bulvarı No: 4 Odunpazarı, Eskisehir 26040, Turkey; Cardiology Department, University Hospitals Plymouth NHS Trust, Plymouth, UK; Department of Cardiology, Rigshospitalet—Copenhagen University Hospital, Copenhagen, Denmark; Carol Davila University of Medicine, Romanian Scientists Academy, Bucharest, Romania; Department of Cardiology, KU Leuven, Leuven, Belgium; Department of Internal Medicine III, Innsbruck Medical University, Innsbruck, Austria; Department of Cardiology, National and Kapodistrian University of Athens, Athens, Greece; Department of Cardiology, KH Hietzing, Vienna, Austria; Department of Cardiology, Hadassah-Hebrew University Medical Center, Jerusalem, Israel; Department of Cardiology, Institute of Cardiovascular Diseases Prof. C.C. Iliescu, Bucharest, Romania

**Keywords:** Ventricular arrhythmias, Acute heart failure

## Abstract

Patients presenting with or alerting emergency networks due to acute heart failure (AHF) form a diverse group with a plethora of symptoms, risks, comorbidities, and aetiologies. During AHF, there is an increased risk of destabilizing the functional substrate and modulatory adding to the risk of ventricular arrhythmias (VAs) already created by the structural substrate. New VAs during AHF have previously identified patients with higher intra-hospital and 60-day morbidity and mortality. Risk stratification and criteria/best time point for coronary intervention and implantable cardioverter defibrillator implantation, however, are still controversial topics in this difficult clinical setting. The characteristics and logistics of pre-hospital emergency medicine, as well as the density of centres capable of treating AHF and VAs, differ massively throughout Europe. Scientific guidelines provide clear recommendations for the management of arrhythmias in patients with chronic heart failure. However, the incidence, significance, and management of arrhythmias in patients with AHF have been less studied. This consensus paper aimed to address the identification and treatment of VAs that complicate the course of patients who have AHF, including cardiogenic shock.

## Table of contents

Introduction
Issues related to organization
 Pre-hospital organization
  Levels of therapy
  Examples of triage criteria suggesting an advanced level
 Physical set up of intensive cardiac care unit and specialized staff
Pathophysiology and prognosis
 Pathophysiology and causes of ventricular arrhythmias in acute heart failure
 Prognostic implications of premature ventricular complexes in acute heart failure
 Prognostic implications of sustained ventricular arrhythmias in acute heart failure
Management
 Preadmission management
 Assessment of comorbidities
 Correction of reversible causes
 Anti-arrhythmic therapies (drug preferences, effectiveness, and limitations)
 Non-anti-arrhythmic medications (including optimization of acute heart failure treatment)
 Device and interventional therapies to control sustained ventricular arrhythmias in acute heart failure patients
 Management of electrical storm
 Coronary interventions: indications and timing
Conclusion and future perspectives
References

## Introduction

The ubiquity of arrhythmias, both atrial and ventricular, in chronic heart failure (HF) is well established, and their significance and treatment have been studied quite extensively. Scientific guidelines provide clear recommendations for the management of arrhythmias in patients with chronic HF. However, the incidence, significance, and management of arrhythmias in patients with acute HF (AHF) in intensive cardiac care unit (ICCU) have been less studied.

The aim of this document is to address the identification and treatment of ventricular arrhythmias (VAs) that complicate the course of ICCU patients who have AHF, including cardiogenic shock (CS). Emphasis is placed on mechanisms and therapeutic strategies. Controversial issues regarding the management of VAs in this specific population are discussed. Clear messages and advice for frequently encountered situations in clinical practice are provided according to strength of statements (*Table 1*).

**Table 1:** Strength of clinical advice statements

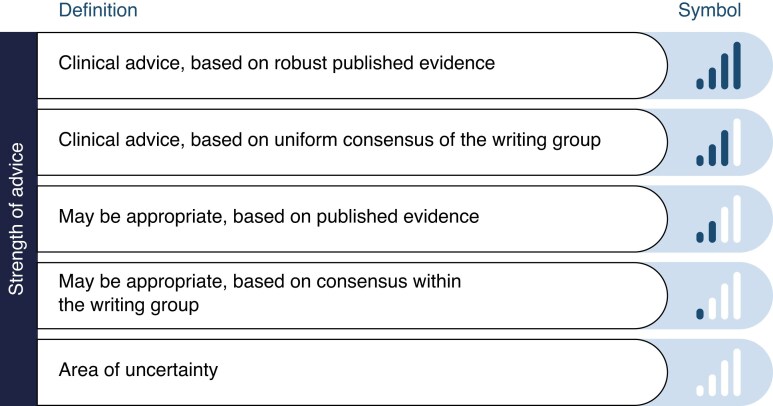

The writing group has been comprised of cardiovascular specialists, each having extensive experience with cardiac arrhythmias and AHF. As the management of these arrhythmias requires a multidisciplinary approach, this scientific statement is representing the consensus of a panel of experts from the Association for Acute CardioVascular Care (ACVC), the European Heart Rhythm Association, and the Heart Failure Association of the European Society (ESC).

## Issues related to organization

### Pre-hospital organization

Patients presenting with or alerting emergency networks due to AHF form a diverse group with a plethora of symptoms, risks, comorbidities, and aetiologies. If this AHF is caused by VAs, the management of the patient is further complicated by the rapid nature of the course of the disease and often the complexity of a patient's medical history. Ideally, thorough information concerning the patient's routine electrocardiogram (ECG), basic echocardiography, and implantable cardioverter defibrillator (ICD) parameters, revascularization history, and managing healthcare professionals should be available when the emergency contact takes place. In addition to the functional status of the patient, this information should be used to help triage the patient and influence the institution to which the patient is transported. If a referral network (‘VT Network’) exists, its organization should provide triage criteria and a specialist on call who could assist with patient placement and pre-hospital anti-arrhythmic and supportive therapy based on the extensive information listed above.

**Figure 1:**
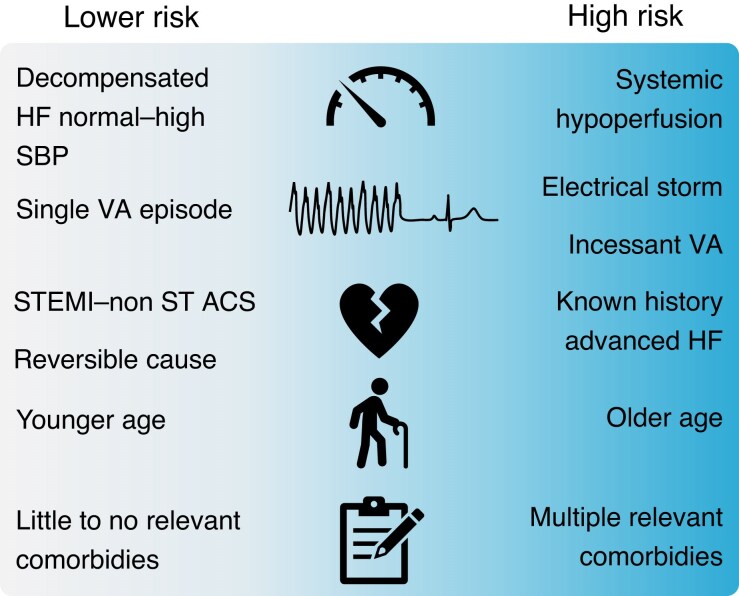
Need for advanced therapies and risk of poor outcome. HF, heart failure; NONSTE-ACS, non-ST elevation acute coronary syndrome; SBP, systolic blood pressure; STEMI, ST elevation myocardial infarction; VA, ventricular arrhythmia.

The characteristics and logistics of pre-hospital emergency medicine, as well as the density of centres capable of treating AHF and VAs, differ massively throughout Europe. Thus, triage, placement, and referral criteria must reflect regional and national circumstances (*Figure 1*).

#### Levels of therapy

1. Intensive cardiac care unit, device interrogation/programming, cath lab
2. Intensive cardiac care unit, specialized HF and arrhythmia service, mechanical circulatory support (MCS)
3. Ventricular tachycardia (VT) ablation (24 h), transplant centre

#### Examples of triage criteria suggesting an advanced level

1. Cardiogenic shock due to refractory VAs
2. History of chronic HF without VAs
3. History of VT ablation in an acute setting

### Physical set up of intensive cardiac care unit and specialized staff

Current cardiology publications detail ICCU layout and technical requirements.^1^ The ICCU should have single (isolation) rooms, a central nurse station, invasive monitoring equipment, a procedure room with X-rays, catering, restrooms, changing rooms, en-suite overnight accommodation for on-call staff, and education and training facilities. When constructing an ICCU, support facilities for relatives and carers, the patient's right to privacy and dignity, noise reduction, and natural light are the major subjects to be pondered.^2^

Any national healthcare system should offer formal acute cardiovascular care education to improve ICCU patient management. The ESC-ACVC core curriculum (CC) for acute cardiovascular care provides a clear education structure. Intensive cardiac care unit cardiologists must meet CC standards. The ESC-ACVC recommends 12 months of full-time training after core cardiology training to become a cardiovascular intensivist. A cardiology director should oversee all ICCUs and be well-versed in acute cardiovascular care and ICCU treatment.^2^

Heart failure patients with ICD and arrhythmias are frail and require HF, electrophysiological (EP), cardiovascular imaging, and palliative care. Successful programmes require physicians, advanced practice providers (APPs), nurses, and technologists. The APPs ensure clinicians from each field are available to address patient needs. Trained cardiovascular implantable electronic device (CIED) interrogators are essential. Nurses or technicians notice device failures, inadequate biventricular pacing, arrhythmias, and HF diagnostic alarms. These concerns must be addressed immediately and brought to the EP and HF teams for a co-ordinated care plan. Nurse co-ordinators can improve patient and provider satisfaction by providing patient education.^3^

## Pathophysiology and prognosis

### Pathophysiology and causes of ventricular arrhythmias in acute heart failure

The generation of VAs in the AHF setting involves a combination of structural and functional substrate (i.e. ischaemia, electrolyte imbalance, etc.) along with modulatory factors. During AHF, there is an increased risk of destabilizing the functional substrate and modulatory factors (i.e. the autonomic nervous system) adding to the risk of VA already created by the structural substrate (i.e. scar/fibrosis, etc., which create conditions for re-entry or abnormal automaticity). The main mechanisms involved in VA during AHF are presented below:

1. Extensive fibrosis (including infarction scars) and EP cell remodelling, including connexins, are classical substrates for re-entry.^4^ Complex circuits of viable myocardium embedded in fibrous tissue result in slow conduction and represent the most important determinant for re-entry.^5^
2. However, increased automaticity determined by acute stretching^6^ of the ventricular myocardium results in VT.
3. Transmural dispersion of refractoriness determined by regional electromechanical feedback and down-regulation of potassium currents contribute to VAs.^7^
4. Acute heart failure can precipitate focal arrhythmias through triggered activity, especially in patients with non-ischaemic cardiomyopathies.^7^
5. Prolongation of the action potential duration is accompanied by early afterdepolarization and the risk of torsade de pointes (TdP).^8^
6. Decreased calcium reuptake by sarcoplasmic/endoplasmic reticulum Ca^2+^-ATPase (SERCA) and increased ryanodine receptor activity and calcium leak may result in increased cytoplasmic calcium with the consecutive sodium calcium exchanger (NCX) hyperactivity and initiation of transient inward current resulting in delayed afterdepolarization and focal arrhythmias.^5^
7. The neuroendocrine hyperactivity [catecholamine storm, increased renin–angiotensin–aldosterone system (RAAS) products] is directly arrhythmogenic and indirectly through electrolytes disturbances (i.e. hypokalaemia and hypomagnesaemia) and vasoconstriction.^9^
8. Myocardial ischaemia provoking AHF or resulting from increased parietal stress could induce VT through multiple direct or indirect mechanisms (enhanced automaticity, alteration in conduction, increased dispersion of repolarization, electrolyte shift, or acidosis). Usually, ischaemia may result in polymorphic VT (different from the scar-induced monomorphic VT) or ventricular fibrillation (VF), and the risk is increased by increased catecholamine drive.
9. Drugs are important contributors to VAs in AHF through direct or indirect pro-arrhythmic effects (*Table 2*).

**Table 2:** Pathophysiologic mechanisms of ventricular arrhythmias in acute heart failure

Structural and functional factors	Arrhythmogenesis mechanism
Electrophysiologic remodelling Action potential prolongation Altered Ca^2+^ reuptake and SR leak Altered K^+^ currents Increased automaticity Abnormal cell coupling	Early afterdepolarization—focal Delayed afterdepolarization—focal Increased dispersion of repolarization Automatic tachycardias Re-entry Re-entry
Structural and functional remodelling Fibrosis and scar Chamber stretch Ventricular dilatation Hypertrophy Ischaemia	Re-entry Automatism Bundle branch re-entry Multiple mechanisms Multiple mechanisms
Neurohormonal mechanisms Increased adrenergic tone Increased RAAS activation Electrolyte abnormalities	Multiple mechanisms and enhancing arrhythmia propensity of other mechanisms
Drugs interactions Anti-arrhythmics, antibiotics, anti-fungal, psychoactive Sympathomimetic drugs Phosphodiesterase inhibitors^10^ Diuretics Digoxin	QT prolongation Increased VT/VF risk Triggered activity, ischaemia Electrolyte disturbances Delayed after depolarization

RAAS, renin–angiotensin–aldosterone system; SR, sarcoplasmic reticulum; VF, ventricular fibrillation; VT, ventricular tachycardia.

### Prognostic implications of premature ventricular complexes in acute heart failure

Premature ventricular complexes (PVCs) may be a complication and a cause of HF. Premature ventricular complexes and non-sustained VAs are common in patients with chronic HF, and in severely reduced ejection fraction are associated with a higher risk for sudden cardiac death (SCD).^11,12^ Persistent PVCs may also result in cardiac remodelling and tachycardia-induced cardiomyopathy.^13^ In rare cases, VF may be triggered by short-coupled trigger PVCs.^14^

Acute heart failure predisposes to PVCs via many mechanisms, including increased filling pressures, elevated sympathetic tone, exogenous catecholamines, ischaemia, and inflammation.^15^ In the EuroHeart Failure survey, VAs were commonly identified in 2%.^16^

As sporadic and short-lasting VAs often remain undetected in AHF, the prognostic implications and the optimum management of PVCs in AHF remain uncertain. New atrial and VAs during AHF have previously identified patients with higher intra-hospital and 60-day morbidity and mortality.^17^ However, left bundle branch block (BBB), non-sustained VT, and frequent PVCs were predictive of SCD in patients with AHF on multivariable analysis.^18^

### Prognostic implications of sustained ventricular arrhythmias in acute heart failure

Sustained VAs occur much less commonly than PVCs and non-sustained VT in patients presenting with AHF, with an estimated incidence of ≤5%.^19^ Although data on the occurrence of VA in AHF are scarce, the negative prognostic impact has been shown in multiple studies showing an increased risk for SCD in these patients.^20–22^ However, in the majority of patients with AHF and systolic dysfunction, the co-existence of sustained VA infers a worse prognosis and often warrants protection with an ICD as an adjunct to medical HF therapy. The exact effect of sustained VA on prognosis in AHF remains undetermined, as many patients are nowadays protected with an ICD in primary prevention, and the presence of sustained VA is not included in any of the clinical risk scores in AHF.^23,24^

## Management

### Preadmission management

Patients with AHF should get appropriate therapy and be promptly transferred to the nearest hospital, preferably with an ICCU (‘time-to-treatment’ concept). In the pre-hospital environment, patients with AHF benefit from non-invasive monitoring, such as heart rate (HR), respiratory rate (RR), blood pressure, peripheral oxygen saturation (SpO_2_), and ECG. According to the 2021 ESC Guidelines for the diagnosis and treatment of acute and chronic HF, oxygen therapy is recommended for SpO_2_ < 90% and non-invasive ventilation is required for respiratory distress.^25^

In all patients with palpitations related to suspected VAs or recurrent ICD shocks should a 12-lead ECG be taken for both spontaneous rhythm and VT. The initial ECG is needed to distinguish ventricular from supraventricular tachycardias (SVTs) and to localize the VT origin site. Even if VAs stop before the ECG is taken, the resting ECG can reveal structural heart disease (SHD), acute coronary syndrome (ACS), channelopathies, possible triggers, and PVC morphology as indicators of VT source or exit.^26^

Haemodynamically unstable or unconscious patients with wide complex tachycardia (WCT) need electrical cardioversion and often advanced cardiac life support (ACLS). In some patients, electrical cardioversion alone could re-establish the haemodynamic stability. Patients with haemodynamically stable monomorphic WCT can undergo vagal manoeuvres. Adenosine can be given if this fails to stop the tachycardia. Adenosine stops atrioventricular-node-dependent SVT with BBB or pre-excitation and rare idiopathic VAs. The next step is anti-arrhythmics. Ventricular arrhythmias must be electrically cardioverted if anti-arrhythmic drugs (AADs) fail.^26^

Implantable cardioverter defibrillators should be interrogated promptly to evaluate arrhythmia burden and determine whether ICD therapies were appropriate and effective or whether VA outside therapy zones is detectable. The ICDs can stop VA with anti-tachycardia pacing (ATP) or an internal shock.^26^

**Figure 2:**
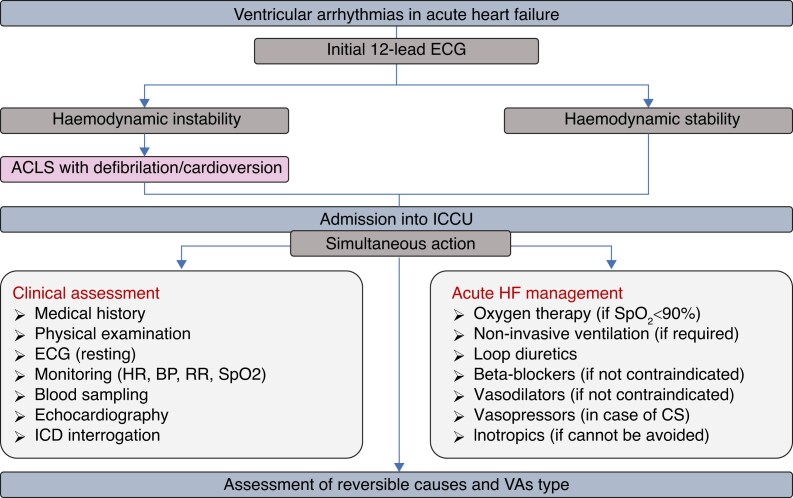
Initial approach to ventricular arrhythmias in acute heart failure. ACLS, advanced cardiac life support; BP, blood pressure; CS, cardiogenic shock; ECG, electrocardiogram; HR, heart rate; ICCU, intensive cardiac care unit; ICD, implantable cardioverter defibrillator; RAAS, renin–angiotensin–aldosterone system; RR, respiratory rate; SpO_2_, pulse oximetry.

Additional diagnostic steps entail echocardiography to evaluate left ventricular (LV) function and exclude SHD and laboratory tests to rule out reversible pro-arrhythmic factors, including electrolyte disturbances.^26^ Initial approach to VAs in AHF is summarized in *Figure 2*.

### Assessment of comorbidities

In an ageing population, co-existing cardiovascular as well as non-cardiac conditions constitute major risk factors for the incidence of both AHF and VAs. However, age and several relevant comorbidities may also affect patients’ outcomes and prognosis.^25,26^ A list of conditions and their clinical relevance are given in *Table 3*.

**Table 3:** Correlations of cardiac and non-cardiac comorbidities with outcomes of ventricular arrhythmia and acute heart failure

Comorbidity	Impact on VA	Impact on AHF
Coronary artery disease	Increased incidence and mortality	Increased incidence and mortality
Valvular heart disease	Increased incidence and mortality	Increased incidence and mortality
Hypertension	Increased incidence	Increased incidence and mortality
History of stroke/TIA	Increased incidence	Increased incidence and mortality
Diabetes mellitus	Unknown	Increased incidence and mortality
Thyroid disorders	Increased incidence	Increased incidence
Obesity, frailty, sarcopenia	Increased mortality	Increased mortality
Kidney dysfunction and electrolyte imbalances	Increased incidence and mortality	Increased incidence and mortality
Chronic pulmonary disease	Increased mortality	Increased mortality
Peripheral vascular disease	Increased incidence and mortality	Increased incidence and mortality
Infection	Increased incidence	Increased incidence
Cancer	Increased mortality	Increased mortality

AHF, acute heart failure; TIA, transient ischaemic attack; VA, ventricular arrhythmia.

Accordingly, history and diagnosis of these conditions are of utmost importance at the onset of AHF symptoms to treat or ameliorate potential triggers, but also to decide about the intensity of possible interventions and potential therapy restrictions. The advice of the writing group is to thoroughly collect information on patient history and related medical reports as quickly as possible. An accurate medical examination, ECG, laboratory analysis, and possibly emergent ultrasound and X-ray analyses are mandatory thereafter. Special emphasis should be placed on acutely modifiable risk factors, such as hypertension, anaemia, kidney failure, and electrolyte disorders, as treatment of these conditions may be able to acutely improve patients’ status and outcomes. A management plan including end-of-life decisions needs to be designed based on this information according to patient status, comorbidities, as well as treatment options and has to be adjusted to the individual will and directives.^26^

Several clinical scores, such as the Sequential Organ Failure Assessment (SOFA) score^27^ and others, have been validated in AHF with similar performance.^28^ Some of these are also available electronically and are helpful in the acute setting to quickly assess patients’ status and prognosis.

### Correction of reversible causes

Reversible causes of VT/VF are common in patients with AHF (*Table 4*). Electrolyte disturbances, particularly hypokalaemia in patients on chronic loop diuretic treatment, should be identified and corrected immediately, as this may reduce the arrhythmic burden significantly. A plasma potassium (K) of 4.0–5.0 mmol/L should be aimed.^29^ Hypomagnesaemia is particularly common in patients with co-existing hypokalaemia and causes TdP, which is most often reversible upon intravenous (i.v.) magnesium administration.^30^ In patients with TdP without other obvious causes, such as bradycardia or QT interval prolonging drugs,^31^ magnesium administration may be pursued even in patients with normal levels of magnesium, as plasma levels may not correctly reflect tissue concentrations.^32^

**Table 4:** Reversible causes of VAs in AHF and what to do for correction

Factor	Corrective actions	Comments
Electrolyte disturbances		
Hypokalaemia	Potassium i.v.	Hypomagnesaemia could be the cause for refractory hypokalaemia
Hypomagnesaemia	Magnesium i.v.	Attempt even with normal K-Mg if TdP
Metabolic acidosis	Sodium bicarbonate	No clear evidence for the effect of medication
Myocardial ischaemia	Revascularization	No clear evidence for the effect of revascularization
Excessive neurohormonal stimulation		
Intrinsic	Optimize volume status, support circulatory status (consider temporary MCS), mild to deep sedation/intubation	
Extrinsic/iatrogenic	Reduce inotrope dose or switch inotrope	Beta adrenergic drugs most prone, but all inotropes may induce VAs
Pro-arrhythmic drugs	Remove pro-arrhythmic drugs	Often Class 1C and Class III (except Amiodarone). Class IV is contraindicated. If digoxin, consider digitalis antibody treatment

AHF, acute heart failure; i.v., intravenous; K, potassium; MCS, mechanical circulatory support; Mg, magnesium; TdP, torsade de pointes; VAs, ventricular arrhythmias.

Metabolic acidosis worsens cardiac function and raises the risk of VF in patients with AHF and hypoperfusion or renal dysfunction. In AHF patients with metabolic acidosis, it is crucial to evaluate and address the root cause thoroughly. Limited data exist on acute correction of metabolic acidosis in the complex metabolic environment of AHF. In a clinical trial, correcting metabolic acidosis with sodium bicarbonate did not reduce 7- or 30-day mortality.^33^

While myocardial ischaemia can be the cause of VT/VF in the setting of AHF, the effect of urgent revascularization is not well documented. However, in patients with ongoing myocardial ischaemia and recurrent VT/VF, the timing of revascularization is discussed in another section below.

Neurohormonal stimulation is invariably present in AHF and may be a cause of VT/VF. Optimization of volume status may alleviate the stimulation and prevent further VAs. Exogenic excessive neurohormonal stimulation caused by adrenergic agonists, such as dopamine, dobutamine, and epinephrine, is a common cause of arrhythmia in AHF and should be managed by reducing inotrope doses or an attempt to switch to another inotrope. Drugs considered to be pro-arrhythmic must be discontinued.

### Anti-arrhythmic therapies (drug preferences, effectiveness, and limitations)

Anti-arrhythmic drug may be used for the termination, suppression, or prevention of recurrent VAs during AHF. The choice of AAD initiation should be based on arrhythmia type, its clinical impact, and parallel treatment for AHF. This should be carefully balanced with possible detrimental side effects of AAD, as these may exhibit cardio-depressant effects, particularly in the setting of diminished LV function. Patients with AHF were excluded from most AAD trials; possible efficacy and safety are mainly to be inferred from studies in patients with HF with reduced ejection fraction (HFrEF) and VA.

Intravenous amiodarone is the drug most used to control VA in the setting of AHF. It has been shown to be effective for the acute treatment of VA requiring repeated cardioversions and in suppression of ventricular ectopy in patients with depressed LV function.^34–37^ Intravenous administration may be associated with a decline in cardiac output, in particular when a large bolus is administered in a short period of time.^38^ Prolonged bolus administration was reported to be without detrimental haemodynamical effects.^34^ The internationally accepted protocol is to load with 150 mg over the first 10 min (15 mg/min), which is followed by 360 mg over the next 6 h (1 mg/min) and to maintain infusion with 540 mg over the remaining 18 h (0.5 mg/min).

Treatment with beta-blockers (BBs), often added to amiodarone, is of importance as elevated sympathetic tone is a factor in the pathophysiology of VA and HF. Discontinuation of BBs at admission for AHF has been associated with poor outcomes.^39^ The timing of novel beta-blocker initiation should depend on haemodynamic status, considering benefits and potential risks. Patients with HFrEF with an electrical storm (ES) have shown benefit from short acting (esmolol) or non-selective (propranolol) beta-blockers.^40,41^

Class I AAD are generally considered contraindicated in HFrEF because of pro-arrhythmic and negative inotropic effects. They may, however, be used in specific situations. Despite its cardio-depressant effects,^42^ mexiletine may be used in addition to amiodarone in long-term management to prevent VT recurrence in HFrEF.^43^ Lidocaine (Class IB) has shown little effect in sustained VA and may only have a role in patients with acute ischaemia after amiodarone and BBs have failed. Procainamide has been shown to be superior compared with amiodarone and lidocaine for conversion of VAs in small trials but is contraindicated in patients with AHF.^44,45^ Finally, quinidine has been proposed for the treatment of polymorphic VAs in short-coupled TdP (pseudo torsade)^46^ and as bail-out therapy for patients with refractory monomorphic VAs.^47^ Of note, Class III AADs (dronedarone and sotalol) with the exception of amiodarone are also formally contraindicated in HF, and Class IV AADs (non-dihydropyridine calcium channel blockers (CCB))) are contraindicated because of strong negative inotropy.^48,49^

### Non-anti-arrhythmic medications (including optimization of acute heart failure treatment)

**Figure 3:**
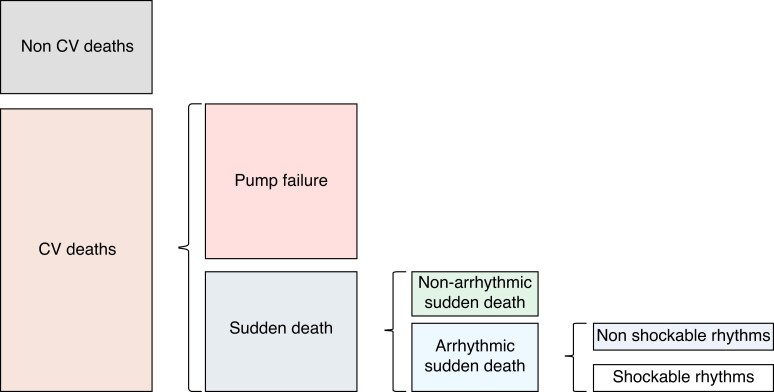
Mechanisms leading to sudden cardiac death. CV, cardiovascular; SCD, sudden cardiac death.

Guideline-directed medical therapies (GDMTs), BBs, angiotensin-converting enzyme inhibitors (ACEis)/angiotensin receptor blockers (ARBs)/angiotensin receptor neprilysin inhibitors (ARNIs), mineralocorticoid receptor antagonists (MRAs), sodium–glucose co-transporter 2 inhibitors (SGLT2is) can inhibit neuro-hormonally mediated VAs, and they may also mitigate the mechanisms involved in the occurrence of other events, such as asystole, electromechanical dissociation, and terminal bradyarrhythmia.^50^ The complexity of the mechanisms leading to SCD (*Figure 3*) likely explains why ICDs do not prevent many SCDs and why neurohormonal antagonists can prevent SCD even in patients who have an ICD in place.^51^

For *de novo* AHF patients with HFrEF, GDMTs should be introduced early during pre-discharge phase in stable patients.^52^ A rapid up titration process is advised, since in STRONG-HF trial a high intensity care strategy was not associated with the increased risk of VAs.^53^

Despite a strong effect of total mortality reduction, ACEi did not significantly decrease the risk of SCD.^54^ In a sub-analysis from PARADIGM-HF,^55^ sacubitril/valsartan-reduced SCD risk regardless of ICD use. Beta-blockers^56^ and MRAs^57^ substantially decrease the risk of SCD and the incidence of malignant VAs. The anti-arrhythmic effects of SGLT2i therapy for patients with HF are uncertain. In the most recent meta-analysis,^58^ including 11 randomized controlled trials (RCTs), SGLT2i therapy in patients with HF was associated with a significantly lower incidence of SCD when compared with placebo (RR: 0.68; 95% confidence interval: 0.48–0.95), irrespective of ischaemic or non-ischaemic HF aetiology or background use of GDMTs. Notably, the meta-analysis did not demonstrate a reduction in the incidence of arrhythmic events, including VAs, in patients receiving SGLT2i therapy. It is possible that the SGLT2i effect on SCD is driven by a reduction in the incidence of non-shockable rhythms, such as pulseless electrical activity and asystole, mechanisms that ICD would not treat.

In patients already taking BBs, ACEi/ARB/ARNI, and MRAs, any effort should be done to continue these therapies, unless major contraindications are present. However, in patients with recurrent VTs or ES associated with haemodynamic instability, ACEi or ARNI should be temporarily stopped and reintroduced after the treatment of VAs. Similarly, BBs with vasodilator properties (Carvedilol) should be replaced with BBs without alpha-1 activity (Metoprolol or Bisoprolol).

Although there is no robust evidence, it may be appropriate to stop SGLT2i in patients with CS and metabolic acidosis or in patients with AHF and hypoglycaemia.

Intravenous inotropic agents (dopamine, dobutamine, milrinone, and levosimendan) may be associated with severe VAs in patients presenting with AHF,^59^ and 2021 ESC Guidelines for the diagnosis and treatment of acute and chronic HF recommend that these medications be given for ‘lowest doses for the shortest possible duration’.^25^ In general, the risk increases with higher doses and longer duration^25,50^ but pro-arrhythmic effects may occur at any dose.^25^ Electrolyte abnormalities (hypokalaemia and hypomagnesaemia), ischaemic aetiology, presence of scar, and inflammation potentiate the pro-arrhythmic risk associated to i.v. inotropes.^60^ During i.v. inotrope administration, continuous ECG monitoring is mandatory, and K and Mg levels should be monitored at 12–24 h.^61^ Weaning of i.v. inotropes should be attempted whenever possible.

### Device and interventional therapies to control sustained ventricular arrhythmias in acute heart failure patients

There are no randomized clinical trials comparing the various MCS devices or ablation strategies in the setting of CS and concomitant VAs. Observational data exist to support the role of MCS in this setting, both to improve haemodynamic stability and suppress VA.^62,63^ However, detailed descriptions of arrhythmia management beyond haemodynamic stabilization are lacking in these studies. In patients requiring catheter ablation (CA), the merits and risks of pre-emptive MCS in high-risk patients have been demonstrated,^64–68^ and recommendations regarding the use of MCS within this setting are included within the latest expert consensus document on CA of VAs.^69^ In contrast, data surrounding the use of bail-out MCS during periprocedural decompensation are poor and do not demonstrate a benefit on procedural outcomes and survival.^64,65,70^

Data specific to CA of VA in patients with AHF is limited. Retrospective single-centre analysis of patients with combined drug refractory VA and AHF undergoing CA demonstrated suppression of VA and survival to discharge in 80%.^71^ Other VA ablation studies have often included subsets of patients with AHF, but interpretation of this data regarding outcomes is limited.^72^ Ballout *et al.*^73^ retrospectively analysed clinical outcomes from prospectively collected data of bail-out VA ablation in a critically unwell population with CS and inability to wean MCS due to refractory VAs despite optimization of AAD therapy. The data present bail-out VA ablation as a viable option with good acute procedural success and subsequent weaning from MCS in most patients.

Management of patients with AHF and sustained VA requires a multidisciplinary approach involving the HF team, intensivists, electrophysiologists, and cardiac surgeons.^74^ Mechanical circulatory support may be appropriate for stabilization of CS combined with or secondary to refractory VA. The choice of MCS should be made based on available expertise. Mechanical circulatory support may result in the cessation of VA. In cases where refractory VA prevents stabilization on MCS and AAD alone or precludes weaning from MCS, bail-out VA ablation may be appropriate. Ongoing work, including prospective and randomized trials, is needed in this area.

### Management of electrical storm

An ES is a common occurrence in patients with ICD. It is defined as three or more episodes of sustained VAs within 24 h, requiring either ATP or cardioversion/defibrillation. Each event must be separated by at least 5 min. In patients with AHF, ACLS including external cardioversion is advised. Mild-to-moderate sedation may be appropriate to reduce psychological distress and decrease pro-arrhythmogenic sympathetic tone after achieving haemodynamic stability. Medical, device, and interventional therapies in sustained VAs are discussed in other sections. According to the 2022 ESC Guidelines for the management of patients with VAs and the prevention of SCD, ICD therapy optimization/reprogramming should be considered in the scenario of repeated ICD discharges and deep sedation/intubation may be considered for patients who do not respond to drug treatment.^26^ Overdrive pacing may be appropriate in patients with AHF with slow incessant monomorphic VT and patients with PVC-induced polymorphic VT at lower HRs.

### Coronary interventions: indications and timing

**Figure 4:**
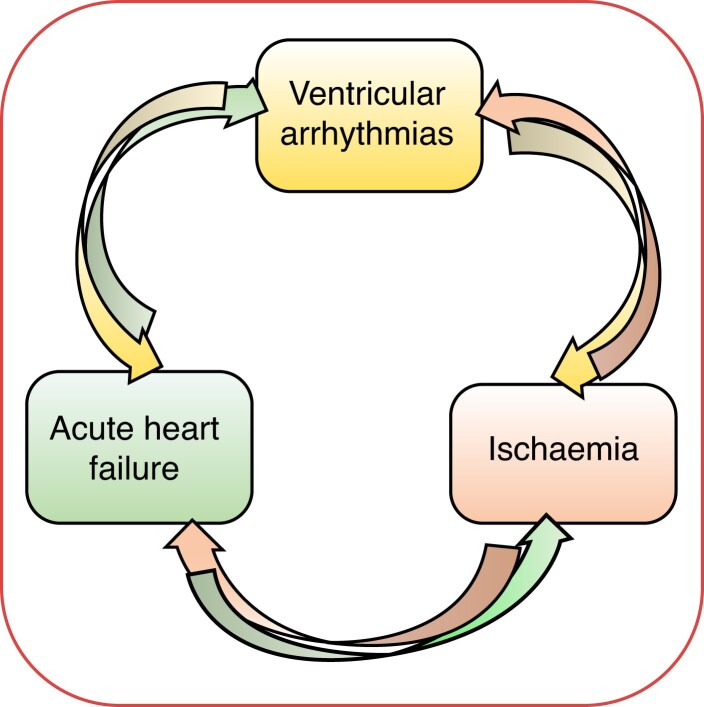
Complex cycle of deterioration during myocardial ischaemia.

Ventricular arrhythmias can arise from ischaemia in the context of AHF or ACS. Conversely, VAs can also lead to both AHF and ischaemia. Thus, accurately identifying regional myocardial ischaemia as the primary cause of this complex cycle of deterioration (*Figure 4*) is challenging but crucial for effective management in certain cases.

**Figure 5:**
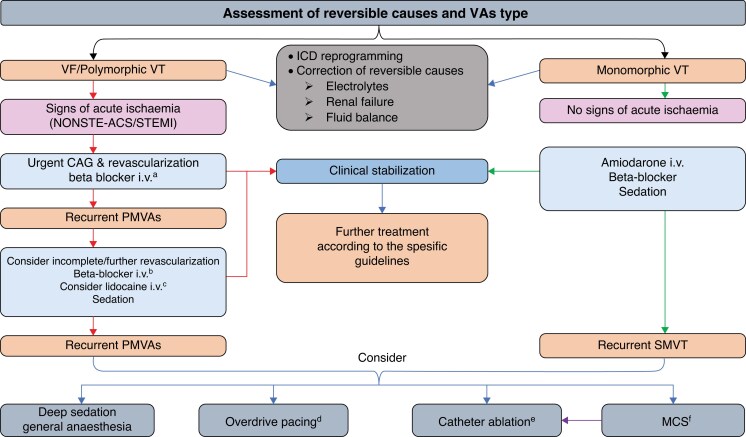
Management of ventricular arrhythmias in acute heart failure. ^a^/^b^Beta-blocker if not contraindicated, i.v. non-selective or short acting beta-blocker preferred. ^c^Lidocaine in cases with acute ischaemia. ^d^Overdrive pacing in polymorphic ventricular arrhythmia with bradycardia or slow incessant ventricular arrhythmia. ^e^Catheter ablation in sustained monomorphic ventricular tachycardia or polymorphic ventricular arrhythmia induced by similar premature ventricular contraction. ^f^Mechanical circulatory support in cases with cardiogenic shock despite optimal medical therapy and to facilitate catheter ablation if indicated. ICD, implantable cardioverter defibrillator; MCS, mechanical circulatory support; NONSTE-ACS, non-ST elevation acute coronary syndrome; PMVAs, polymorphic ventricular arrhythmias; SMVT, sustained monomorphic ventricular tachycardia; STEMI, ST elevation myocardial infarction; VA, ventricular arrhythmia; VF, ventricular fibrillation; VT, ventricular tachycardia.

**Figure 6:**
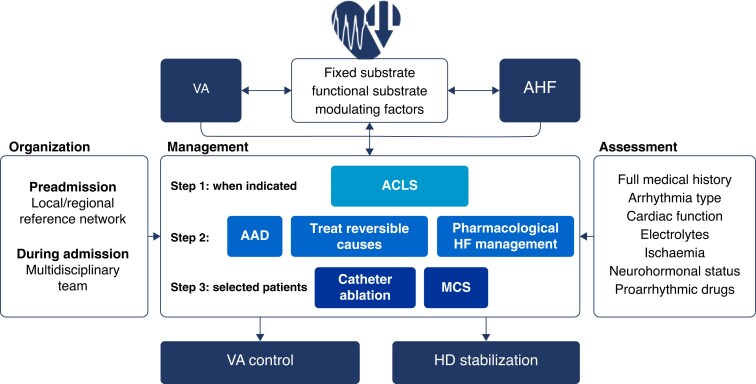
Summary of management of ventricular arrhythmias in acute heart failure. AAD, anti-arrhythmic drug; ACLS, advanced cardiac life support; AHF, acute heart failure; HD, haemodynamic; MCS, mechanical circulatory support; VA, ventricular arrhythmia.

Ventricular fibrillation/polymorphic VT commonly occurs in the presence of acute ischaemia, while monomorphic VT generally indicates an existing arrhythmogenic substrate^75^ and ischaemia does not contribute to its development.^76^ Therefore, distinguishing between VF/polymorphic VT and monomorphic VT is the initial step in determining the need for coronary interventions. Subsequently, evaluating 12-lead ECG for ischaemic changes should be the next step. In cases of ST elevation myocardial infarction (STEMI) and non-ST elevation ACS (NSTE-ACS), urgent reperfusion (<2 h) and before percutaneous coronary intervention i.v. beta-blockers are advised if not contraindicated.^26^ Particularly in recurrent monomorphic VAs, after correcting the other reversible causes, CA is advised.^26^ Management of VAs in AHF is summarized in *Figures 5* and *6*.

## Conclusion and future perspectives

Ventricular tachycardia is still challenging in the setting of AHF. As highlighted in this document, a multidisciplinary therapeutic approach is often required to treat VT in this clinical setting. The options for pharmacological therapies are limited, and EP procedures in the setting of AHF carry substantial procedural risk and mortality. *Table 5* summarizes the clinical advice of the writing group. The 2022 ESC Guideline for the management of VA and SCD provides guidance for ACVC physicians.^26^ The ESC Guideline encompasses several diagnostic and treatment algorithms. In particular the place and timing of MCS in the management of VAs in the setting of AHF should be further studied. Risk stratification and criteria/best time point for ICD implantation, however, are still controversial topics in this difficult clinical setting. Importantly, in patients with haemodynamically not-tolerated sustained VT or VF during the acute phases of cardiac decompensation, ICD implantation before hospital discharge is advised. This aggressive concept of immediate ICD implantation needs to be balanced against the possibility of using wearable ICDs. Thus, several knowledge gaps remain, unfortunately.^77^

**Table 5:** Clinical advice in the patients with ventricular arrhythmias and acute heart failure

Statement	Strength of clinical advice	Section
In AHF, there is insufficient evidence that PVCs or non-sustained VAs in the absence of sustained VAs are of prognostic relevance. This task force advises to evaluate patients individually in order to administrate an additional treatment for PVCs and non-sustained VAs	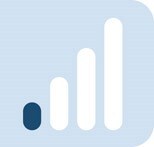	Prognostic implication of PVC in AHF
At admission a 12-lead ECG of both sinus rhythm and VAs is beneficial for further patient management	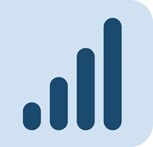	(Pre)admission management
In ICD carriers with AHF and recurrent ICD therapies, prompt ICD interrogation at admission is advised to exclude inappropriate therapy and optimize settings	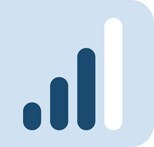	(Pre)admission management
For patients with AHF and haemodynamically unstable sustained VAs, ACLS with external cardioversion/defibrillation is advised	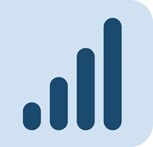	(Pre)admission management
This task force advises patients with AHF and VAs to be transferred to the nearest hospital, when available this should be a centre equipped with an ICCU	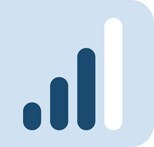	(Pre)admission management
Patients with VAs with suspected AHF benefit from non-invasive monitoring of rhythm, RR, BP, SpO_2_, and prompt echocardiography to assess causes of AHF	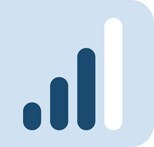	(Pre)admission management
In patients with VAs and AHF, early echocardiography to assess biventricular function and valvular abnormalities is advised	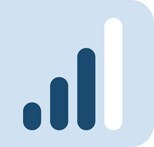	(Pre)admission management
Initial pharmacological management of patients presenting with AHF and VAs includes simultaneous efforts to suppress VAs, relieve congestion, and relieve hypoperfusion	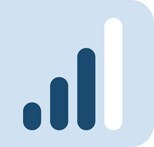	(Pre)admission management
In patients with AHF and VAs, use of i.v. inotropic agents should be avoided. If it is mandatory to, it is advised only in the smallest possible dose and for the shortest possible time. Norepinephrine is advised to be the first choice in patients with CS	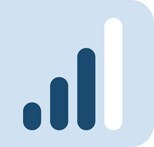	(Pre)admission management, non-AAD management
Medical history and diagnosis of (co)morbidities are crucial to treat or ameliorate potential triggers, but also to decide about the intensity of possible interventions and potential therapy restrictions	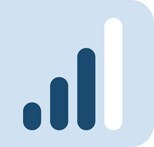	Assessment of comorbidities
Assessment of possible acutely modifiable risk factors, such as hypertension, anaemia, kidney failure, and electrolyte disorders, is advised as treatment of these conditions may be able to acutely improve patients’ status and outcomes	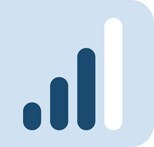	Assessment of comorbidities
In patients with AHF and VAs, electrolyte disturbances, particularly hypokalaemia in patients on chronic loop diuretic treatment, should be identified and corrected immediately	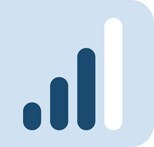	Correction of reversible causes
In patients with co-existing TdP and hypokalaemia, administration of i.v. magnesium is advised	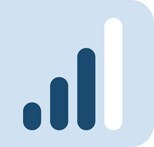	Correction of reversible causes
It is advised to consider neurohormonal stimulation, an important causative and maintaining factor for recurrent VA in all patients with AHF and VAs	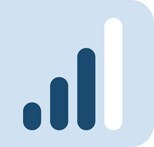	Correction of reversible causes
Loop diuretics are advised in decompensated AHF and VAs to ameliorate congestion and elevated sympathetic tone as maintaining factor for VA	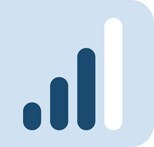	Correction of reversible causes
Sedation may be beneficial in patients presenting with AHF and VA to alleviate excessive neurohormonal stimulation as underlying or maintaining factor for VA	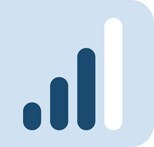	Correction of reversible causes
Exogenic neurohormonal stimulation caused by adrenergic/inotropic agonists is a common cause of VAs in AHF and should be managed by reducing inotrope doses or a switch to another inotrope	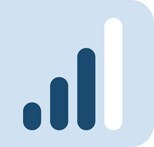	Correction of reversible causes
Deep sedation or general anaesthesia may be appropriate to alleviate neurohormonal stimulation as maintaining factor for VAs in patients with AHF and recurrent VAs after initial pharmacological treatment and correction of reversible causes	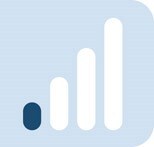	Correction of reversible causes
In patients with AHF and recurrent polymorphic VT/VF with clinical evidence of STEMI/NON-STEMI, urgent CAG and revascularization is advised	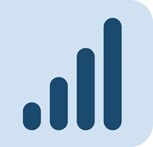	Timing of coronary intervention
In patients with AHF and recurrent polymorphic VT/VF with unknown cause after initial assessment, early CAG and revascularization may be appropriate	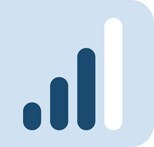	Timing of coronary intervention
In patients with AHF and recurrent monomorphic VT without overt clinical signs of cardiac ischaemia, CAG and revascularization may not be beneficial for the prevention of recurrent VAs	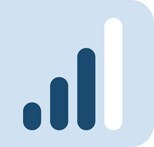	Timing of coronary intervention
Intravenous amiodarone is as the first line of AAD treatment in patients presenting with AHF and VAs	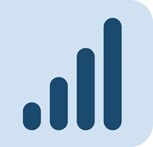	AAD management
In patients with AHF and VAs, prolonged infusion of amiodarone may be appropriate after a first fast charging bolus administration. However, second fast bolus should be avoided as it has been associated with a decline in cardiac output.	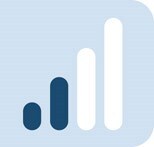	AAD management
It is advised to avoid discontinuation of BB treatment at admission for AHF and VAs	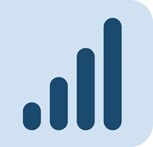	AAD management, non-AAD management
If the haemodynamic status permits, the use of short acting or non-selective BBs may be appropriate for patients with AHF who experience recurrent VAs after being managed with amiodarone	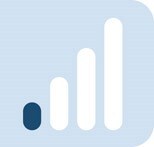	AAD management
In patients with AHF and VAs, quinidine may be appropriate in the treatment of polymorphic VAs in patients with chronic ischaemic heart disease and as bail-out therapy for patients with refractory monomorphic VAs	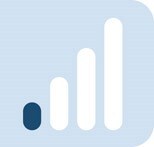	AAD management
In patients with AHF and recurrent VA with haemodynamic instability, ACEi, ARB, and ARNI should be temporarily stopped	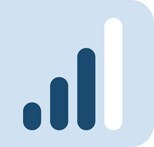	Non-AAD management
In patients with AHF and VAs, it may be appropriate to stop SGLT2i in patients with CS and metabolic acidosis or in patients with AHF and hypoglycaemia	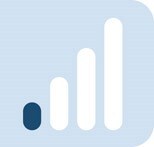	Non-AAD management
In patients with AHF and ES/incessant VAs due to monomorphic VT, CA is advised after failed pharmacological treatment	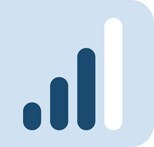	Device and interventional therapies to control sustained VAs in AHF patients
In patients with AHF and recurrent polymorphic VT/VF, CA may be appropriate if VAs are triggered by a unifocal PVC and pharmacological treatment has failed	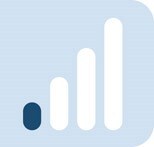	Device and interventional therapies to control sustained VAs in AHF patients
In patients with AHF who cannot be weaned form MCS due to recurrent monomorphic VA or polymorphic VA induced by a similar PVC, CA may be appropriate	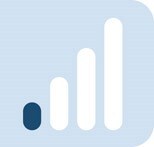	Device and interventional therapies to control sustained VAs in AHF patients
Overdrive pacing may be appropriate in patients with AHF with slow incessant monomorphic VT and patients with PVC-induced polymorphic VT at lower heart rates	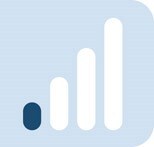	Device and interventional therapies to control sustained VAs in AHF patients
MCS may be appropriate for patients experiencing intractable VAs and CS despite receiving optimal medical therapy and if they are not candidates for CA	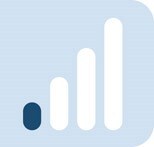	Device and interventional therapies to control sustained VAs in AHF patients
MCS may be appropriate for patients experiencing intractable VAs and CS despite receiving optimal medical therapy to facilitate CA if not otherwise possible	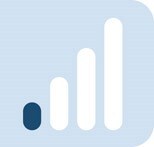	Device and interventional therapies to control sustained VAs in AHF patients

ACLS, advanced cardiac life support; AHF, acute heart failure; BP, blood pressure; CAG, coronary angiography; CS, cardiogenic shock; ECG, electrocardiogram; ES, electrical storm; ICD, implantable cardioverter defibrillator; ICCU, intensive cardiac care unit; MCS, mechanical circulatory support; PVCs, premature ventricular complexes; RR, respiratory rate; VAs, ventricular arrhythmias; VF, ventricular fibrillation; VT, ventricular tachycardia.

## Data Availability

All data are incorporated into the article and its online supplementary material.
